# Time to act on childhood obesity: the use of technology

**DOI:** 10.3389/fped.2024.1359484

**Published:** 2024-02-16

**Authors:** Debora Porri, Letteria Anna Morabito, Paola Cavallaro, Elisa La Rosa, Alessandra Li Pomi, Giorgia Pepe, Malgorzata Wasniewska

**Affiliations:** Unit of Pediatrics, Department of Human Pathology of Adulthood and Childhood, University of Messina, Messina, Italy

**Keywords:** childhood obesity, childhood obesity prevention, mobile health, telemedicine, new technology

## Abstract

Childhood obesity is rapidly increasing worldwide and there is an urgent need to implement treatment and prevention programs. Over the last decade, in addition to increasing rates of childhood obesity, we have also observed rapid technological and digital development. The Covid-19 pandemic has largely contributed to both expansions but has also allowed an opening towards a broader vision of medicine, through new therapeutic opportunities such as mobile healthcare. The digital and technological delivery of obesity prevention and treatment programs can represent an innovative tool to support children and families to overcome some limitations and barriers such as the accessibility of programs that prevent them from adopting healthy lifestyle changes. This review aimed to summarize the impact of different digital interventions for children and adolescent affected by obesity.

## 1 Introduction

Especially in the last two decades, the rapid development of urbanization, socioeconomic growth and modernization have contributed to a dramatic increase in obesity rates, favoring unhealthy lifestyles characterized by physical inactivity and an unbalanced diet (1).

The WHO estimates that the obesity percentage has nearly tripled worldwide since 1975 (2).

To date, even childhood obesity can be defined as a global epidemic that could lead to various comorbidities in adult life such as diabetes, hyperlipidaemia, hypertension or hepatic steatosis (3, 4) and it seems that the situation is destined to get worse.

Furthermore, the recent Coronavirus (COVID-19) epidemic has led to a worsening of an already dramatic situation: social isolation, changes in food-seeking behaviour and more sedentary domestic activities have led to the adoption of unhealthy habits by the whole family, contributing significantly to a rapid increase in childhood obesity rates (5).

The need for strategic intervention programs is increasingly important, especially from the early stages of obesity development. Tailored-phase interventions based on modifiable factors such as adherence to the Mediterranean diet, minimizing the consumption of processed and high-calorie-dense foods, reduction of sedentary lifestyle by promoting

physical activity, regularization of sleep, and consequently the control of body mass index (BMI) and weight are mandatory (6).

Interestingly, despite the dramatic consequences, the needs dictated by the social isolation policies essential to contain the pandemic, have also resulted in a “catalyst” of changes necessary for health monitoring, encouraging innovations in healthcare at an unprecedented level (7, 8). Digitalization and mobile device technology advancements have changed communication and offer new opportunities to improve healthcare and patient management.

To date, there are several tools in the medical-healthcare context, both for patient monitoring and remote clinical evaluation. “Digital health” is a new term that includes concepts such as mobile health (mHealth) and telemedicine where clinicians use new mobile apps, smart devices, sensors or wearables, and health information technologies, in order to ensure or improve patient involvement and follow-up and improve medical care, from prevention to treatment (9).

Lifestyle interventions to manage and prevent obesity have proven effective in weight loss and most of the new apps, websites and devices, are aimed at encouraging changes in the patient's lifestyle, through advice, reminders, stimuli, food diaries, self-monitoring of diet and physical activity and motivational support (10).

Precisely in 2020, the year of the pandemic, the National Institute for Health Research (NIHR) funded a study that produced a website and a mobile app named “HelpMeDoIt!” aimed at supporting weight loss intervention for adults affected by obesity (11) by strategies such as goal setting, progress monitoring and social support.

To date, growing evidence highlights several barriers to weight loss intervention success in children and adolescents; beyond the difficulties of directly involved subjects, families and caregivers could present numerous issues (12); beyond the difficulties of directly involved subjects, families and caregivers could present numerous issues; factors driving attrition across weight management programs could be represented by lack of parental time, little time at home, difficulty in following clinical follow-ups, low socioeconomic status but also lack of awareness in the family and parental misperception of the child's weight (13).

Considering the global burden of childhood obesity, it is necessary to implement innovative interventions and tools for the prevention and treatment of excess weight in children, taking into account the various obstacles that can compromise success. The role, involvement and education of parents could be included in new strategies aimed at improving various lifestyle habits in children, in order to reduce childhood obesity and overweight (14, 15).

## 2 Objectives

To date, most studies using digital technology have been conducted on adults themselves. However, evidence has shown that it could also be useful during childhood and adolescence.

This narrative review aims to meticulously investigate and comprehensively describe new digital strategies and technological opportunities aimed at preventing and combating childhood obesity. It stands out for its thorough compilation of diverse technological tools.

Furthermore, we conducted an analysis of the effectiveness and limitations of each tool described by the literature in the context of childhood obesity management and obesity prevention.

## 3 Methods

In order to obtain a broad perspective on the use of new technological innovations in the prevention/treatment of childhood obesity and their characteristics and strengths, each author identified and reviewed the most appropriate published studies (original articles and reviews in English) on this topic.

The following keywords were used to search for papers published from 2013 up to September 2023: pediatric obesity; childhood obesity; obesity in children; pediatric overweight; childhood overweight; overweight in children; obesity prevention; obesity treatment; app; new technology; digital health; smartphone; smartphone app; mobile health; digital follow up, telemedicine.

Electronic databases PubMed, Scopus, EMBASE, and Web of Science were used for research. All records were collected and the drafted preliminary version was discussed among authors, then the final version was then recirculated and approved by all the co-authors.

## 4 New strategies to act on obesity: the use of apps and digital health

Digital health is a broad category that comprises digital medicine (DM) and digital therapeutics (DTx): DTx represents a collection of services in the healthcare and wellness sectors; examples of DTx applications include digital or wearable devices and artificial intelligence (AI).

The Digital Therapeutics Alliance (DTA) defines DTX as “providing evidence-based therapeutic interventions to patients guided by software to prevent, manage or treat a medical disorder or disease. They are used independently or together with drugs, devices or other therapies to optimize patient treatments and health outcomes” (16).

DM uses algorithms, software or hardware products supported by evidence, to measure or intervene in human health. Digital diagnostics, digital biomarkers, and remote patient monitoring devices are some examples of digital medicines.

DM includes DTx, and both concepts are part of the more general and comprehensive term “digital health”.

The first approach to digital health was started by the American Thomas Bird who coined the term “telemedicine” to indicate “the practice of medicine without the usual physical confrontation between doctor and patient, using an interactive multimedia communication system” (17).

The WHO Global Observatory for Electronic Health defines mobile health (mHealth) as “medical and public health practice supported by mobile devices, such as mobile phones, patient monitoring devices, personal digital assistants, and other wireless devices” (18). Digital health can help make health systems more efficient and sustainable, enabling them to deliver good quality, affordable and equitable care. Digital health has been shown to improve the quality of care, increase access to health information and services, and promote positive changes in health behaviors in order to prevent the onset of acute and chronic diseases (18).

Over the last decade, smartphones have become the most common portable electronic device used in the world and the recent and rapid increase in the sector of technological innovations has led to the development of numerous digital tools (apps) effective in enhancing treatments for the management of some chronic diseases (19, 20). In recent years, evidence has shown that telephone services are known as the most efficient and cost-effective method for patient follow-up, reducing the dropout rate (21–23).

Children and adolescents are increasingly exposed to multiple solicitations from the digital world, not only traditional televisions and desktop computers but above all the latest mobile devices such as smartphones and tablets and this seems to have increased since the beginning of the COVID-19 pandemic (24). The relationship between exposure to multimedia screens and obesity has been widely studied, highlighting a clear direct correlation between the daily hours of screen time and the prevalence of overweight and obesity in children (25, 26). Furthermore, the literature highlights an association between screen time in children and adolescents and the adoption of unhealthy lifestyle habits such as increased calorie intake, ultra-processed foods and sweets, sedentary behavior, increased weight and overall reduced quality of life (27).

Numerous studies have evaluated the effects of reducing children's exposure to screen media on weight gain, finding encouraging results (28, 29).

However, in the current era of “digital health”, the direction has changed. The future of stopping the ever-increasing pediatric obesity rates focuses precisely on the use of technology and screens.

The widespread use of mobile electronic devices, in particular, smart devices (such as mobile phones and tablet Personal Computers), has accelerated in the last decade opening new possibilities and strategies aimed at curbing the obesity epidemic expansion; in order to do this, it's essential to act with both prevention strategies and specific and personalized treatments.

The heterogeneous nature of the induced stimuli and functions of digital health tools enables adaptations and upscaling of interventions to fit larger population groups, and input will likely be different depending on the stakeholders.

mHealth interventions allow accessible interaction, frequent contact, and data monitoring through mobile applications, which improve short-term outcomes. These systems have been broadly classified into 5 types: mobile applications (apps), web-based tools, text messages, portable monitoring devices/personal digital assistants (PDAs) and pedometers (30).

Each of these digital stimuli or supports has recently been used to prevent weight gain in children or as an aid in the treatment of childhood obesity and overweight. One of these tools, or more than one, may prove effective in combination with standard treatment, or a particular digital support may be more useful in adolescence rather than school age. Parents have also often been encouraged to use technology to support their children suffering from obesity or overweight.

Technology can be of great help but it is necessary to clarify the times, the end-users and the most suitable tools depending on age in order to target a modern strategy to prevent or reduce childhood obesity.

In the following paragraphs, the use of principal digital and mHealth tools to fight the increase in pediatric obesity rates will be discussed.

### 4.1 Telemedicine and text messages

Telemedicine is the application of electronic information and communication technologies to provide and support a patient's clinical health status (16, 31).

In the last years its role has been explored in managing children and adolescents with obesity with encouraging results (32): it provides innovative access to health care and ensures important support and comprehensive care for children and their families based on their health and biopsychosocial needs (33).

Data extracted from randomized control trials (RCTs) and systematic reviews have shown that telemedicine has been introduced into the management of obese paediatric patients through various technology-enhanced measures: telephone contact for obese children (34, 35) or other types of interventions, such as remote service delivery (36), video conferencing (37), short message service (SMS), and text messaging (38, 39).

Changes in BMI *z*-Score (BMIz) are the most frequently studied primary outcome (32), and its modification is often compared between the intervention and control groups (40–43). Some studies reported a minimal, but statistically significant reduction in BMIz (around 0.1 points or less) following interventions that incorporated telehealth (37, 40, 41, 44, 45), while other authors (41) found no modifications of BMI between the telemedicine group and the in-person usual care visits group.

Improvement in eating habits (35, 39, 40), physical activity, and patient satisfaction were often investigated as secondary outcomes.

The use of text messaging programs has been associated with changes in health behaviours (fruit and vegetable consumption, low total caloric intake physical activity, and time spent on screen) (35, 39, 40) and with a significant reduction on drop out rate (46) in obese children.

However, a wide variability in results was observed, probably due to the heterogeneity of study protocols and the type of interventions applied. It is still unclear whether there is a technological approach associated with greater BMI reduction or significant improvement in eating habits.

Good feasibility and fidelity characterise all telemedicine interventions on weight reduction. Most studies, especially RCTs, have shown that high retention rates (e.g., 80%–100% among telemedicine groups) and high attendance at telemedicine appointments are the most commonly cited strengths of these tools (35, 40, 43, 47).

Parents' and patient's satisfaction is another important strength of the telemedicine approach (37, 42), and this element probably influences the attested higher adherence to weight management programs, also in obese children from rural populations with poor access to tertiary care (47).

Despite the positive results shown by RCTs on eating behaviors and patient and family satisfaction, telehealth and telemedicine programs have some limitations. We are unable to confirm the absolute validity of this approach on goals such as weight and BMI reduction, especially in long-term observation. Available studies do not report overlapping information on the duration of follow-up, which is highly variable, and the magnitude of the effects of interventions is difficult to determine.

In addition, high differences among the samples examined, including heterogeneity in ethnicity, socioeconomic status, and education, compromise the results and are a possible weakness of the available studies (32). Some studies included primarily white/Caucasian non-Hispanic participants (35, 40, 48) or did not assess the impact of ethnicity (49) on the results shown. These elements limit the ability to generalize results to different populations (32).

Another important limitation is the high cost-effectiveness ratio. Research has shown that telemedicine software is expensive to maintain, and the availability of powerful Internet connections, software, and equipment is essential. These elements could limit access to telemedicine and telehealth tools in patients of low socio-economic status or living in rural areas, where such equipment can be most beneficial (43).

A possible solution could be the combination of telehealth intervention with in-person visits, that seems to be associated with positive results (50).

### 4.2 Mobile app

The use of remote technologies such as mobile phones and mobile apps is gaining ground in childhood obesity management and has been explored in several weight loss or obesity prevention programs.

The use of the mobile apps was evaluated both as a single strategy and in combination with traditional treatments and literature seems to show a good efficacy level, even if studies had different features; for instance, Lei et al. (51) recently performed a historical observational analysis including 2,825 adolescents (mean age 14.4–2.2 years) affected by overweight or obesity who used a mobile app (The MetaWell), without any type of face-to-face interaction, providing participants with low-calorie meal plans and encouraging them to reduce sedentary behaviors. Participants were asked to report data at three checkpoints (42, 60, 90, and 120 days after first accessing the app) and authors observed statistically significant differences in weight reduction at each time point. In a fairly recent trial comparing standard care and standard care with the addition of digital support (the OBEST app) conducted by Likhitweerawong (52) on 77 children and adolescents with severe obesity, it was found that the intervention group was significantly related to the increase in the trend of healthy eating behaviors—less frequent consumption of fast food, as observed by the odds ratio, despite authors didn't find a significant difference in weight change between subjects who had access to digital support and those who didn't have it. Also Hagman (53) and colleagues revealed the effectiveness of a digital support system in combination with clinical visits; in their study, authors evaluated the effectiveness of a pediatric obesity lifestyle treatment with digital support compared to randomly selected matched controls from a clinic register, which received only standard care. Treatment focuses on lifestyle modification to reduce the degree of obesity by improving dietary habits and increasing physical activity.

Interesting, differently to Likhitweerawong and colleagues (52) their digital support also focuses on encouraging parents to be responsible for the outcome of the treatment (53): the digital support system, as well as providing guidance related to diet and lifestyle as well as previously mentioned strategies (51, 52), provides frequent communication between clinical staff and families.

Authors (53) performed a stratified analysis, revealing that the treatment effect was superior among patients with digital support compared with standard care in both males and females, young children and adolescents.

Family-based treatment (FBT) is a weight management approach for children with obesity that involves in-person behavioral counseling with parent-child dyads and has been shown to be an effective approach in terms of weight loss (54) and the parent's/caregivers' involvement is also foreseen by several mHealth technologies; for instance, Tripicchio et al. (55) presented an overview of the development and preliminary testing of iByte4Health, a mHealth obesity prevention program delivered via smartphone that integrates parent and child-facing content aimed at allowing parents and children to work together towards goals, and engage in behavior change toward healthy habits. It's important to notice that parents were interested in receiving information across a range of targets related to their child's health, but a primary concern was their own feeding practices. Parents reported an increase in monitoring their children's snack consumption and interestingly reported a reduction in their use of snacks as rewards (55). In addition, there was a slight increase in parent-reported encouragement of child physical activity.

Similarly to Tripicchio and colleagues (55), Alexandrou et al. (56) conducted a study to evaluate the effectiveness of a 6-month parent-oriented mHealth intervention (MINISTOP 2.0 app) aimed at preventing childhood obesity. Authors had previously developed a novel mHealth intervention focused on preschool-aged children, to promote healthy lifestyle behaviors like intake of fruits, vegetables, sweet and savory treats, sweet drinks, moderate-to-vigorous physical activity, and reducing screen time (57).

Researchers recruited 552 parents with a 2.5–3-year-old child who were randomly assigned to the intervention group with mHealth support (*n *= 277) in addition to standard care or control group (*n* = 275) which received only standard care (56).

Children in the intervention group had lower reported intakes of sweet and savory treats, and sweet drinks, as well as less screen time at follow-up compared to the control group. Additionally, parents in the intervention group also reported a higher parental self-efficacy score for promoting healthy lifestyle behaviors at follow-up compared to the control group.

Is important to notice that the parent's misperception of the child's weight could represent a difficult obstacle to overcome in this context. Contrary to previous studies (53, 55–57), Cox and colleagues found a very small parent engagement (58) in their study aimed at testing the feasibility of a new app (FoodT); unfortunately, the majority of parents refused app utilization (40/62, 65%) and the reasons included not only disinterest in using an app, but above all the belief that their child's weight is not related to food choices; therefore, parents felt that other aspects rather than food choices should be taken into consideration, despite their children being followed in a pediatric clinic for the problem of excess weight. However, compared to previous work (53, 55–57) in this study, the average age of the children involved was 15.3 years, so the adolescent's decision-making autonomy may have influenced the parent's choices.

What seems to emerge from recent studies (53, 55–57, 59) is that the positive feedback from parents regarding the use of the app depends on their involvement during the development of this technology.

A recent study also highlighted the involvement of the end user, in particular in those who were personally involved, in this case, a group of teenagers. Martin et al. (60) enrolled a total of 74 adolescents aged 13–16 years from Spain, Italy, and the United Kingdom who participated in the co-design and prototyping processes of the PEGASO Fit for Future (F4F) mHealth intervention for adolescents. Participants were involved in the co-design, refinement, and feasibility testing of mobile apps aimed at encouraging healthy lifestyle–promoting behaviours.

Giorgi Rossi and colleagues (61) described the process for co-creating an app to be installed on parents' mobile phones aimed at preventing childhood obesity in the context of “The BMInforma project”, included in the CoSIE project (Cocreation of Service Innovation in Europe, Call: H2020-SC6-CO-CREATION-2016–2017). Literature on similar cooperation methods is quite extensive, highlighting the essential role of end-users, in order to fill the gap between end-user needs and professional's needs and objectives (62–67).

### 4.3 Web-based tools and social networks

Social media platforms offer a unique opportunity to connect with a wide audience. Public health campaigns and non-profit organizations have successfully used social media to raise awareness about obesity-related issues, provide resources and connect individuals with shared interests. Support groups, forums, and online communities allow individuals to share their experiences, challenges and successes in managing their weight.

Holmberg et al. (68) explored the effects of online information as an obesity treatment in a group of 20 adolescents, aged 13–16. Participants used a screen-recorded computer to demonstrate their search procedures and were interviewed with questions about their experiences of engaging with online information and content regarding food, weight management and health. The interviewer initially aimed to reach a shared understanding of central concepts such as “health,” “nutrition,” “information,” and “social media.” As the interview progressed, it was possible to explore why and how the participants searched for and selected online information regarding food, weight management, and health, as well as how they experienced and evaluated this information. In this study, the adolescent participants reported searching for food and workout information not just to lose weight, but to feel healthy and to perform better in their daily lives. This indicates that adolescents also value these more integrated aspects of health and well-being, not just for losing weight. The adolescents described both encouraging and discouraging experiences using online information and the authors observed that online forums and social networks could suggest healthy meal ideas and provide social support for a behavior change. Conversely, they also documented discouraging experiences, such as feeling disconsolate by fitness models and disappointment over unsuccessful weight loss attempts. In addition, there was a confusing amount of misleading commercial content online and experiences of food marketing in online networks.

This concept has been further investigated by Folkvord et al. (69), whose review evaluated if changing the advertising and media environment could help children and adolescents choose healthy foods instead of unhealthy ones, in order to reduce childhood obesity and improve children's health. By evaluating how adolescents search and choose the information about food, weight management, and health on websites and social networks, the characteristics that were taken into consideration and perceived as trustworthy were the reliability, accuracy and ease of finding the information, together with the presentation format (69). To this end, more colorfully and attractively designed websites and videos were more attractive than articles (69).

Hammersley et al. (70) conducted a randomized controlled trial focused on assessing the effectiveness of an online childhood obesity prevention program called “Time2bHealthy”. This program targeted parents of preschool-aged children (2–5 years old) and aimed to provide them with valuable information and tools for managing their children's weight and promoting healthy habits. The study assessed various outcomes related to dietary habits, physical activity, and weight management. The results indicated promising outcomes, suggesting that the online program had a positive impact on parents’ knowledge and behaviors regarding childhood obesity prevention. However, further research and evaluation are necessary to determine the program's long-term effectiveness and its potential for widespread implementation.

The above-mentioned findings can be of inspiration for clinicians to promote the development of the most suitable technologies for children and parents, and updating pediatric health communication methods in a more appealing way by using a greater variety of distribution systems and referring to reliable websites.

### 4.4 Exergames, serious videogames and immersive gaming

In recent years, videogames have emerged as a novel approach to addressing the growing concern of obesity. Contrary to traditional views of gaming as a sedentary activity, certain videogames have been designed specifically to encourage physical activity and promote healthier lifestyles. The use of these tools, which are widely appreciated by children and adults, can be an effective instrument to combat sedentary lifestyle, increase physical activity, promote knowledge of an adequate lifestyle and therefore combat overweight and obesity (71–73).

Active video games, known as exergames, blend entertainment with physical activity. These games typically involve bodily movements such as dancing, jumping, or performing exercises that mimic real-life activities (71–73). They involve the use of sensors that are worn on the body to capture the movement performed and transform it into action in the videogame's virtual reality. Exergames are designed to elevate heart rate and engage various muscle groups, providing a means for players to exercise while gaming (72–74). Depending on the muscular complex involved in the action, there will be different energy expenditure, with greater energy commitment if the muscles of the lower limbs are used, and a lower energy commitment for the movements of the upper limbs. Through these games it is possible to practice moderate intensity physical activity, equal to walking at a velocity of 5.7 km/h in normal weight children (73).

Thanks to their enjoyment and motivation, exergames are very appealing to children and promote long-term compliance. Involvement increases if specific goals are set, as feelings of pleasure are stimulated through virtual rewards when the child manages to complete the pre-established objective (73).

Calcaterra et al. (71) reviewed the effects of exergames on obesity. Among the video games analyzed were “DDR Dance Dance Revolution”, “Wii Boxing”, “Eyetoy”, “Kinect Sport” and “Pokemon Go”. The authors highlighted the effects of such videogames in increasing physical activity time and intensity, improving body composition, reducing body weight and insulin resistance. Psychological effects have been associated with the use of exergames, such as greater self-esteem, and a better quality of life (72, 73).

Other types of videogames used to fight the obesity epidemic are serious videogames, which are designed to achieve a change in a specific health behavior while entertaining (74).

These games are oriented to nutrition education and dietary change and are based on executive functioning training that regroups higher cognitive processes allowing skills such as choosing healthier food or resisting food temptation (73–75).

Espinosa Curiel et al. (76) described the development of the multidisciplinary serious video game “Food Rate Master” and the effects on 62 children between 8 and 10 years old in Mexico. The results showed that food knowledge and capability of distinguishing between healthy and unhealthy food was better after playing the videogame. In addition, the frequency of unhealthy food intake was lower and parents' perception was positive, observing the improved children's behavioral capacities towards healthy food.

The effects of using serious videogames in preventing childhood obesity were recently evaluated in a systematic review (77). Following an in-depth research, the authors selected 26 studies that analyzed 17 videogames. What they observed was that physical activity increased during the game, even reaching levels of moderate-vigorous physical activity, while no effect on physical activity outside of the game was evident. Furthermore, by acting through cognitive learning strategies, an improvement in knowledge regarding adequate nutrition and the best lifestyle to prevent and treat overweight and obesity was observed. Among the 17 games analyzed, only 2 (Squire Quest! 1 and 2) involved practical activities, for example the preparation of healthy dishes, making the experience not only cognitive but also sensorial, allowing the child to touch, smell and taste healthy food.

Recent advances in virtual reality (VR) technology are offering a new and promising strategy to increase physical activity and fight overweight and obesity. This technology offers immersive gaming experiences that simulate real-world scenarios and environments (78). VR-based games designed for physical activity can engage users in interactive exercises, sports simulations, and adventurous activities, potentially increasing motivation for regular exercise (79, 80). Preliminary studies suggest that VR-based exercise games have the potential to increase physical activity levels and adherence to workout routines. The immersive nature of VR creates an environment that encourages movement and engagement, providing an enjoyable alternative to traditional exercise regimens. On the other hand, there is still insufficient data on the safety and effectiveness of VR in young children (81).

The above-mentioned videogames and immersive games offer gamification strategies for health goals (82). Gamification involves integrating game design elements into non-game contexts, such as health and fitness goals. By incorporating features like rewards, challenges, and progress tracking into health-related apps or games, individuals are motivated to engage in behaviors conducive to weight management and overall well-being. The use of gamification in health interventions has shown promise in promoting behavioral changes related to diet, exercise, and weight management. By providing immediate feedback, creating achievable goals, and fostering a sense of accomplishment, gamified approaches can enhance motivation and adherence to healthy habits (68, 83).

However, it is also necessary to point out that digital media can hide pitfalls. Various persuasive marketing techniques are employed by food brands to improve consumers' attention, not only toward healthy products (84). In contrast to exergames and serious videogames to contrast obesity, there are videogames that promote branded products, typically food and beverages that are high in calories and low in nutritional values, called advergames. This marketing activity is often carried out in a subtle way, so that children do not realize the commercial intent of the game (84).

## 5 Discussion

To date, childhood obesity rates have reached worrying levels (83). There is a growing need to improve strategies and programs to prevent weight gain and address unhealthy eating habits that could lead to childhood obesity or overweight.

It is well-known that the development of obesity in pediatric age is associated with multiple and serious comorbidities, including type 2 diabetes, increased cardiovascular risk and orthopaedics problems. Obese children and adolescents could also experience social marginalization, with possible negative effects on mental health, academic performances and future employment opportunities (83).

Multi component interventions, including both diet and physical activity, are required to combat obesity pandemic successfully. Traditional approaches (nutritional interventions, outpatient in-person visits, outdoor physical activities) are not always well accepted by patients and their families because of a series of limitations, including high costs, transportation difficulties, and lack of provisions.

In recent years, most research has focused on the effectiveness of technology-based interventions in the management of childhood obesity. Data extracted from these studies are often heterogeneous and the results on the outcomes examined (reduction in BMI *z*-score and/or body weight, improving in nutritional habits and physical activity), while encouraging, have not always reached statistical significance (85).

There is also a lack of knowledge about the impact of a full range of technology-based interventions on weight management in already overweight and obese children, especially with regard to long term observation. In this narrative review we aimed at discussing the main digital tools useful to paediatricians, families and directly children or adolescents for weight management and lifestyle changes.

**Figure 1:**
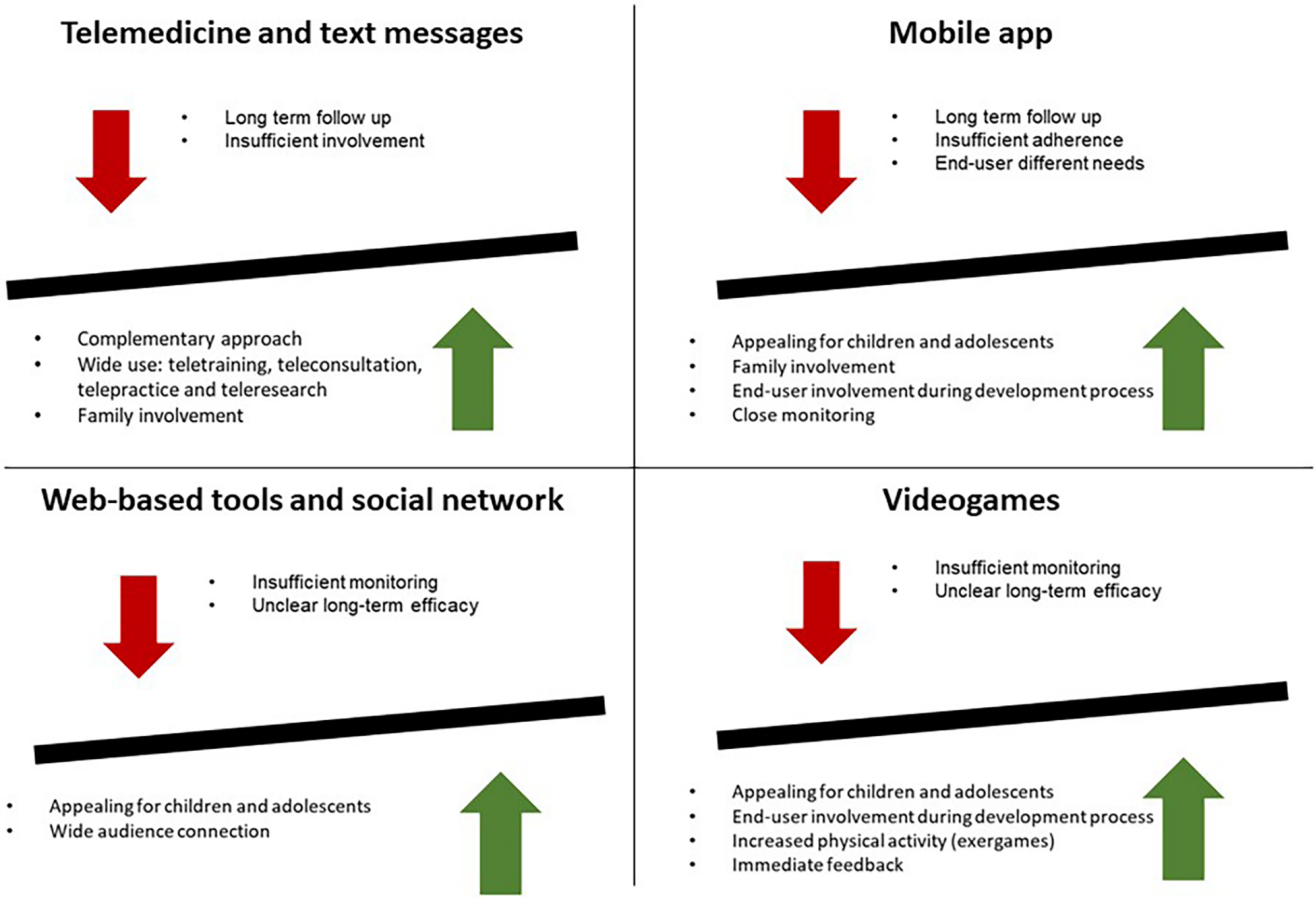
Advantages and limits of new technologies for the prevention and management of childhood obesity.

Most recent studies, especially randomized control trials (Supplementary Table S1), have revealed no significant changes in BMi *z*-score associated with digital health support compared to standard care. However, we are witnessing a clear change in lifestyle habits, towards the improvement of diet, food choices and physical activity, with a reduction in the consumption of junk food and sweet drinks. It is important to note that the follow-up period varied between 6 months and 12 months and maybe not enough to reflect the change in lifestyle on the BMI. Figure 1 schematically summarizes both limits and strengths of the main digital tools used to fight childhood obesity.

What emerges from the literature is the potential of telemedicine and mHealth tools, but it is important to consider some limitations. The need for Internet connections, powerful software and equipment to access telemedicine services (86) is necessary to access digital support services. In addition, the high costs of device ownership and the possible marginalization of some patients because of a scarce attitude toward technology (52) that could influence the eventual return to traditional, in-person visits (86). Finally, the use of telemedicine has provided patients with innovative access to health care and physicians with rapid patient assessment, monitoring, and treatment, but more evidence is needed to ensure the effectiveness of this care approach. Particularly, patient engagement is fundamental to the treatment efficacy and today we are seeing a shift towards more patient-centric competency models (87).

Patient involvement, adherence to behavioural rules and follow-up could be strengthened with some digital stimuli provided by mHealth tools like mobile apps and particularly from videogames and exergames, but what seems to make the difference is the patient engagement during mHealth tool development. Especially for adolescents, their involvement during the development process increases the chances that the mHealth tool will be effective, as it is aimed at finding a compromise between their point of view and the needs and clinical objectives (53, 55–57, 59, 77, 88).

In addition to the adolescents' point of view, it is interesting to note the parents’ point of view on the use of digital health technologies for the management of childhood obesity or overweight.

Family environments play a pivotal role in the context of obesity: it must be protective and anti-obesogenic, especially due to the strong influence of parent's habits on their children's habits. Goa et al. (89) analyzed 14 articles that evaluated parent's experiences and needs in the use of mHealth strategies, such as SMS, telephone, apps, websites, and social media, in the management of overweight or obese children aged 2–10. They demonstrated that technologies are considered a useful tool by parents, but it is important to involve them in the development of mHealth strategies, in order to design suitable tools to meet the family needs. Moreover, it's mandatory to increase parents' awareness and consciousness regarding childhood obesity comorbidities in infancy and later in life.

De-Jongh González and colleagues (90) conducted a randomized control trial exploring “digital phenotype”, meaning typologies of users derived from individuals' patterns of interactions with specific app features (i.e., Unengaged, Socially engaged, Independently engaged, Partially engaged and Fully engaged). Further research in understanding program design elements that may influence participant engagement could be more useful in this area of digital health.

However, it is also necessary to underline that digital media are the children's favorite source of information in the digital age in which we live, and various persuasive marketing techniques are employed by food brands to improve consumers' attention, not only toward healthy products (68). For instance, in contrast to exergames and serious videogames, there are videogames that advertise unhealthy food, called advergames. Advergames are online games that promote branded products, to expose youth to foods and beverages that are typically high in calories and low in nutritional values. This marketing activity is often carried out in a subtle way, so that the child does not realize the commercial intent of the game.

Children and adolescents have always been a special audience, not only due to their developmentally vulnerable, but also because they have always been among the heaviest users of technologies (84).

## 6 Conclusions and future perspective

Supporting technological innovations for weight management is an area of great potential that could overcome barriers such as access, location and cost among people with obesity or overweight. These interventions provided through new digital tools could be very useful for several reasons: first of all, we all now have access to the internet and consequently the number of excluded people would be reduced. In addition, digital tools allow feedback to be provided in real-time, drastically reducing the costs of accessing a visit, and allowing the patient to be followed for a long time. Digital health is a vast area to explore, and new technologies could be very useful in treating and preventing childhood obesity, but it is mandatory to take into account targeting based on age, end users and engagement of the family, especially during the technological development process.

Including end users and empowering them throughout the entire mhealth's development process is useful not only to increase awareness of the obesity problem but also to allow developers to know and meet the user's needs and respond to their specific needs. All childhood obesity prevention or treatment programs should take into account several factors in which the family environment and parents consciousness play a pivotal role.

Clinicians should also consider the very thin limit between the risks and benefits of technology use in children and adolescents.

According to the findings of the most recent RCTs studies (Supplementary Table S1) even if there isn't a significant change on BMI, children behavioral changes are promising, but there is a need for future studies that use larger sample size, longer intervention and follow-up periods, focusing on mHealth tools personalization according to patients needs. To elucidate the potential of technology, measures of engagement and effective satisfaction of requirements are necessary to provide comprehensive and sustainable support for patients and health service providers in order to act on childhood obesity.

In conclusion, sustaining long-term engagement with mhealth technologies designed for health purposes remains a challenge. Strategies to maintain user's interest and motivation over extended periods of time are crucial for ensuring continued participation and benefit.
