# Supplementary figures and images for: Time to act on childhood obesity: the use of technology
Source: Front Pediatr. 2024 Feb 16;12:1359484. doi: 10.3389/fped.2024.1359484 (PMC10904600; doi:10.3389/fped.2024.1359484)

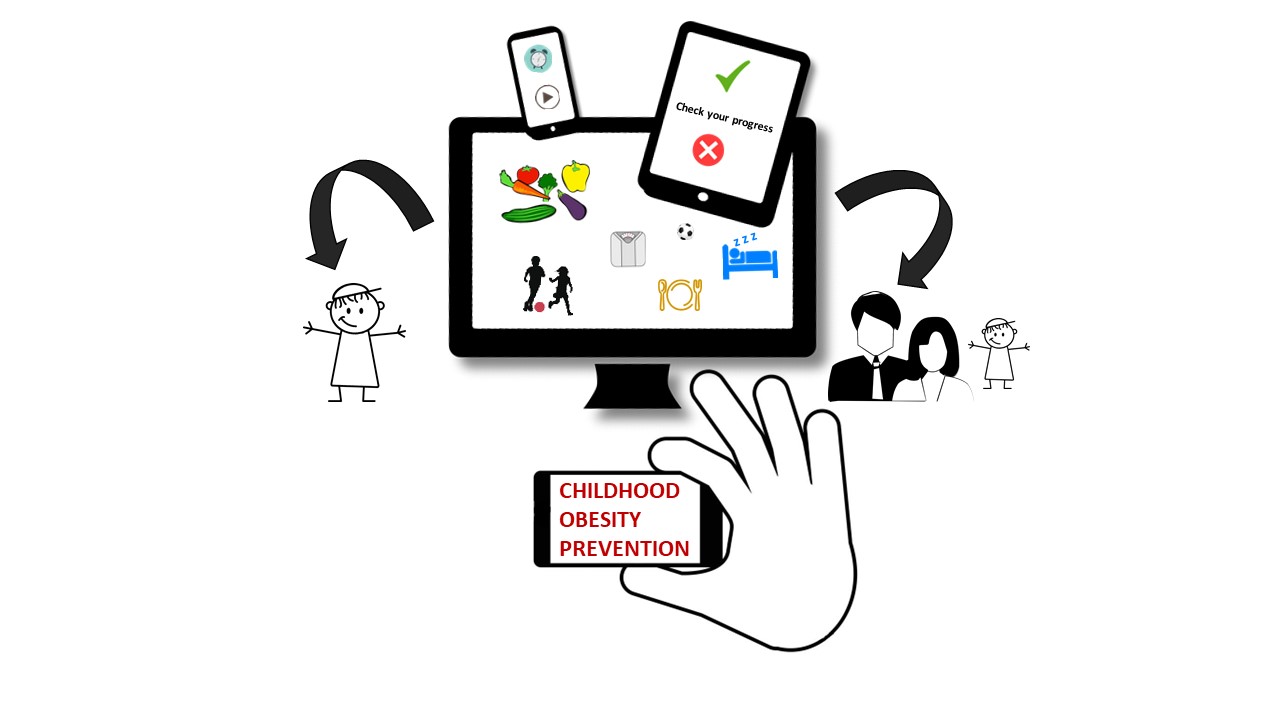

Supplement: Supplementary file 1 [file Image1.jpeg]
