# Supplementary material for: Time to act on childhood obesity: the use of technology
Source: Front Pediatr. 2024 Feb 16;12:1359484. doi: 10.3389/fped.2024.1359484 (PMC10904600; doi:10.3389/fped.2024.1359484)
Supplement: Supplementary file 2 [file Table1.pdf]

Table S1 Most recent RCT evaluating different technologies impact on pediatric obesity management

**Author, year** (Reference number)	**Study design**	**Type of intervention**	**Participants** (number)	**Age** (Range)	**Outcomes**	
					**BMI z score reduction**	**Content and behavioral components**
Bohlin et al 2017	RCT	Phone (Phone coaching vs phone usual care)	40 children	5-15 years	No<br>No difference between groups at the end of treatment (*p* > 0.80)	NA
Chai et al., 2019	RCT	Telehealth (videoconferencing + text messaging + website + online group)	46 children + caregivers	4-11 years	No<br>No statistical differences between the groups	Improving in family/child eating habits: reduced percentage energy from energy-dense nutrient-poor food (*p* =0.038)
Fleischman et al., 2016	RCT Control cross over	Video conferencing<br>(GROUP 1: primary care physician visit + televisits vs GROUP 2: primary care physician visits only for 6 months, then opposite sequence during the second 6 months)	40 children+ caregivers	10-17 years	No differences between the groups during the 12 months follow up. months (*p* = 0.96).<br>Group 1: significant change compared to baseline at each time (6 months : *p* = 0.0006; 12 months: *p* = 0.03).<br>Group 2: significant change compared to baseline at 12 months (*p* = 0.03).	Dietary glycemic load was significantly different from baseline during the obesity specialist treatment (p=0.001 for group 2 at 12 months)<br>High retention rate (90% at 6 months and 80% at 12 months)
Rifas-Shiman et al., 2016	RCT	Phone calls<br>Intervention care: (4 in-person visits and 2 phone calls in the 1st year followed by 2 in-person visit in the 2nd year) vs<br>Usual care visits	445 children (253 intervention group and 192 usual care)	2-6 years	No<br>No differences between the groups during the 2 years follow up (p= 0.06)	No difference in television viewing time, intakes of fast food and sugar sweetened beverages between the 2 groups
Taveras et al.,2017	RCT	Video conferencing or phone-calls or in-person (participant preference) +<br>text messaging	721 children+ caregivers	2-12 years	No<br>No statistical difference between the groups (*p*= 0.39).<br>enhanced usual care plus coaching and enhanced usual care groups both improved	Improvements in children health-related quality of life
Likhitweerawong et al, 2021	RCT	Mobile app (OBEST)<br>Group (1) received standard care combined with the OBEST application<br>Group (2) received only standard care	77 children and adolescents	10-15 years	Yes<br>The magnitude of BMI change in the intervention group was greater than that in the standard care group but did not reach a significant level	The participants in the intervention group had a decreased engaging in fast-food consumption than the standard care and an increase engaging in healthy eating behaviors.
Hagman et a,l 2022	RCT	Digital support system (mobile app) in combination with clinical visit	427 children and adolescents	4-18 years	Yes<br>the mean ± SD change in BMI Z-score in the treatment group was −0.30 ± 0.39 BMI Z-score units and in the standard care group −0.15 ± 0.28, p = 0.0002	NA
Alexandrou et al, 2023	RCT	Digital support system (MINISTOP app) in combination with clinical visit	539 parents and 552 children	2.5-3 years	NO<br>In terms of children's BMI z-score, quantile regression analyses revealed no statistically significant effect at follow-up (50th percentile: 0.0; 95% CI -0.09 to 0.09; and 90th percentile: 0.04; 95% CI -0.03 to 0.11; both p > 0.05).	Statistically significant intervention effects on mean intakes of sweet and savory treats (-6.97 g/day; 95% CI -11.14 to -2.81; *p* = 0.001), sweet drinks (-31.52 g/day; 95% CI -49.05 to -13.98; *p* < 0.001) and average time spent in front of a screen (-7.00 min/day; 95% CI -12.46 to -1.55; *p* = 0.012)<br>Parents in the intervention group reported a significantly higher total PSE (Parental Self-Efficacy) score (0.91; 95% CI 0.26 to 1.55; *p* = 0.006) compared to the control group.

Liu et al, 2022	RCT	App<br>Eat wisely and Move Happily	1392 children	9.6 years	Yes<br>the mean BMI decreased in the intervention group, whereas it increased in the control group; the mean between-group difference in BMI change was -0.46 (95% CI, -0.67 to -0.25; P < .001)	The intervention also improved other adiposity outcomes, dietary, sedentary, and physical activity behaviors, and obesity-related knowledge.
Hammersley et al., 2019	RCT	Online childhood obesity prevention program called “Time2bHealthy”	86 children+ caregivers	2-5 years	No<br>No significant difference in the BMI change between the 2 groups at 6 months post baseline.	The intervention improved dietary intake, child feeding, and nutrition parent self-efficacy.
